# Supplementary material for: PT320, a Sustained-Release GLP-1 Receptor Agonist, Ameliorates L-DOPA-Induced Dyskinesia in a Mouse Model of Parkinson’s Disease
Source: Int J Mol Sci. 2023 Feb 28;24(5):4687. doi: 10.3390/ijms24054687 (PMC10002999; doi:10.3390/ijms24054687)
Supplement: Supplementary file 1 [file ijms-24-04687-s001.zip › ijms-2192126-supplementary.pdf]

Supplementary

PT320, Sustained-Release GLP-1 Agonist, ameliorates L-DOPA-Induced Dyskinesia in a Mouse Model of Parkinson's Disease

	1	2	3	4
**Axial**	Sustained deviation of head/neck by at least 30°	Sustained deviation of head/neck by at least 60°	Torsion of neck/upper trunk by at least 90°	Torsion of neck/upper trunk by >90° causing the mouse to lose balance
**Limb**	Tiny displacement of the forepaw around a fixed position	Larger movement causing visible displacement of the whole forelimb, usually to/from snout.	Large displacement of the whole forelimb with visible engagement of shoulder muscles	Vigorous limb displacement, crossing over the midline of the body
**Abnormal paw movements**	Mice stood on both hind paws, with one front paw moving repeatedly up and down along the surface of the cylinder wall	Mice stood on both hind paws, with both front paws moving repeatedly up and down along the surface of the cylinder wall	Mice stood on their hind paws and repeatedly touched the surface of the cylinder wall with one hind paw.	Mice stood on their hind Paws close to the wall of the cylinder moving both front paws as noted above and repeatedly lifting the hind paws up and down
**Standing**	Keep standing 1~5 seconds	Keep standing 6~10 seconds	Keep standing 11~15 seconds	Keep standing > 20 seconds

Supplementary Figure S1.

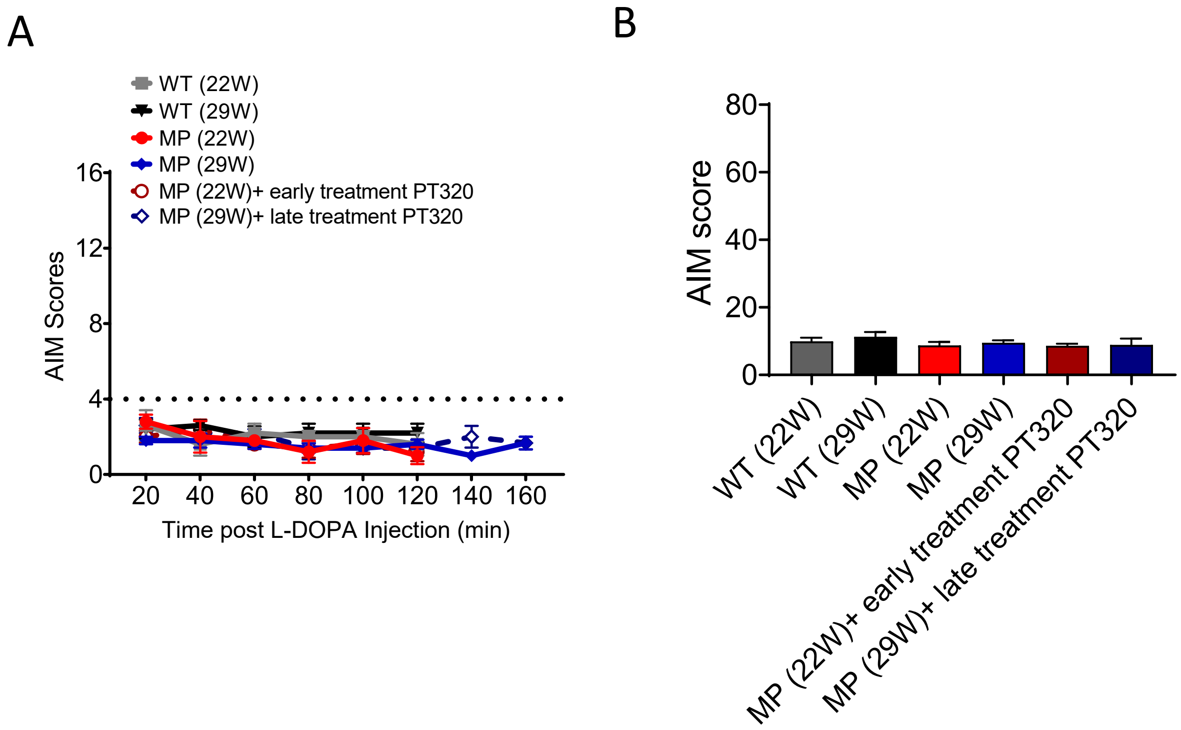

Supplementary Figure S2.

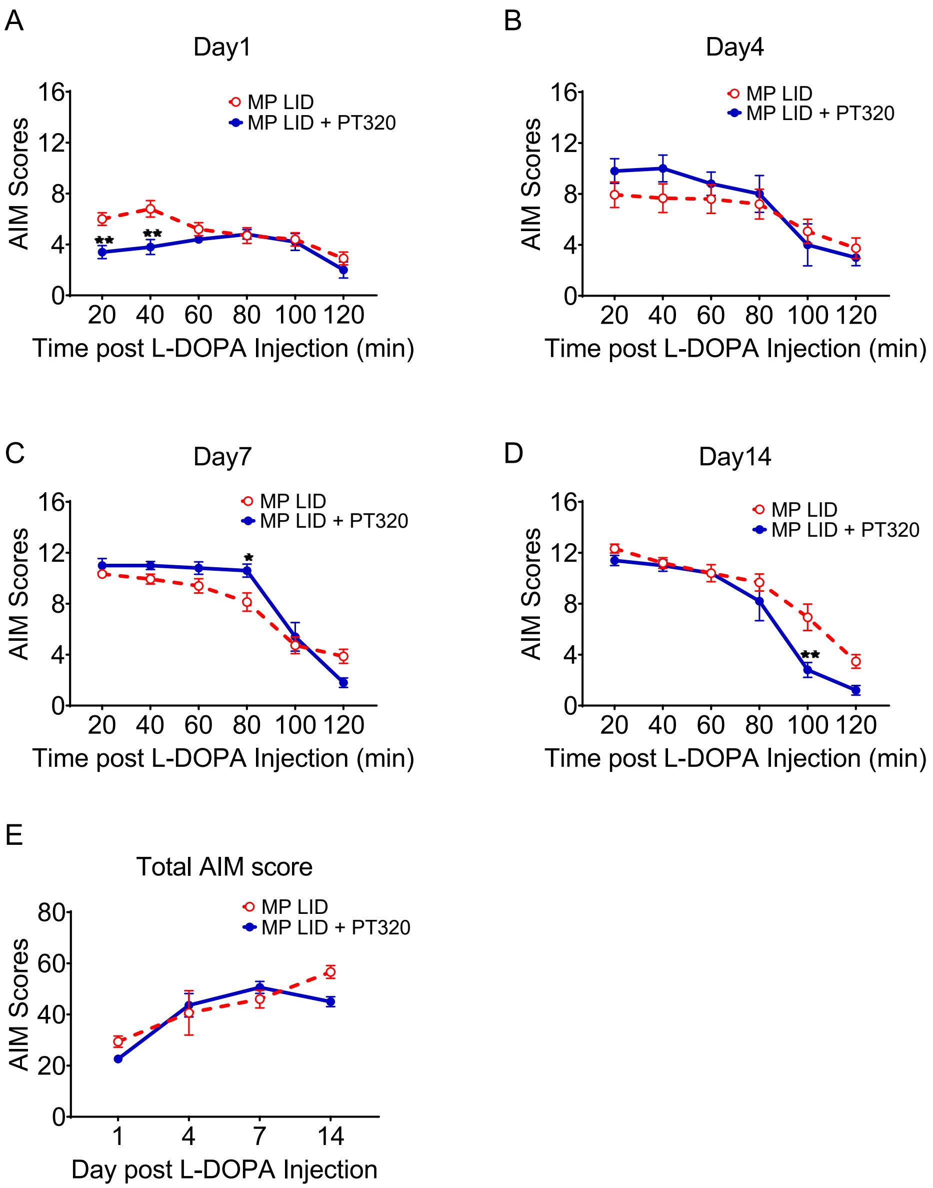

Supplementary Figure S3.

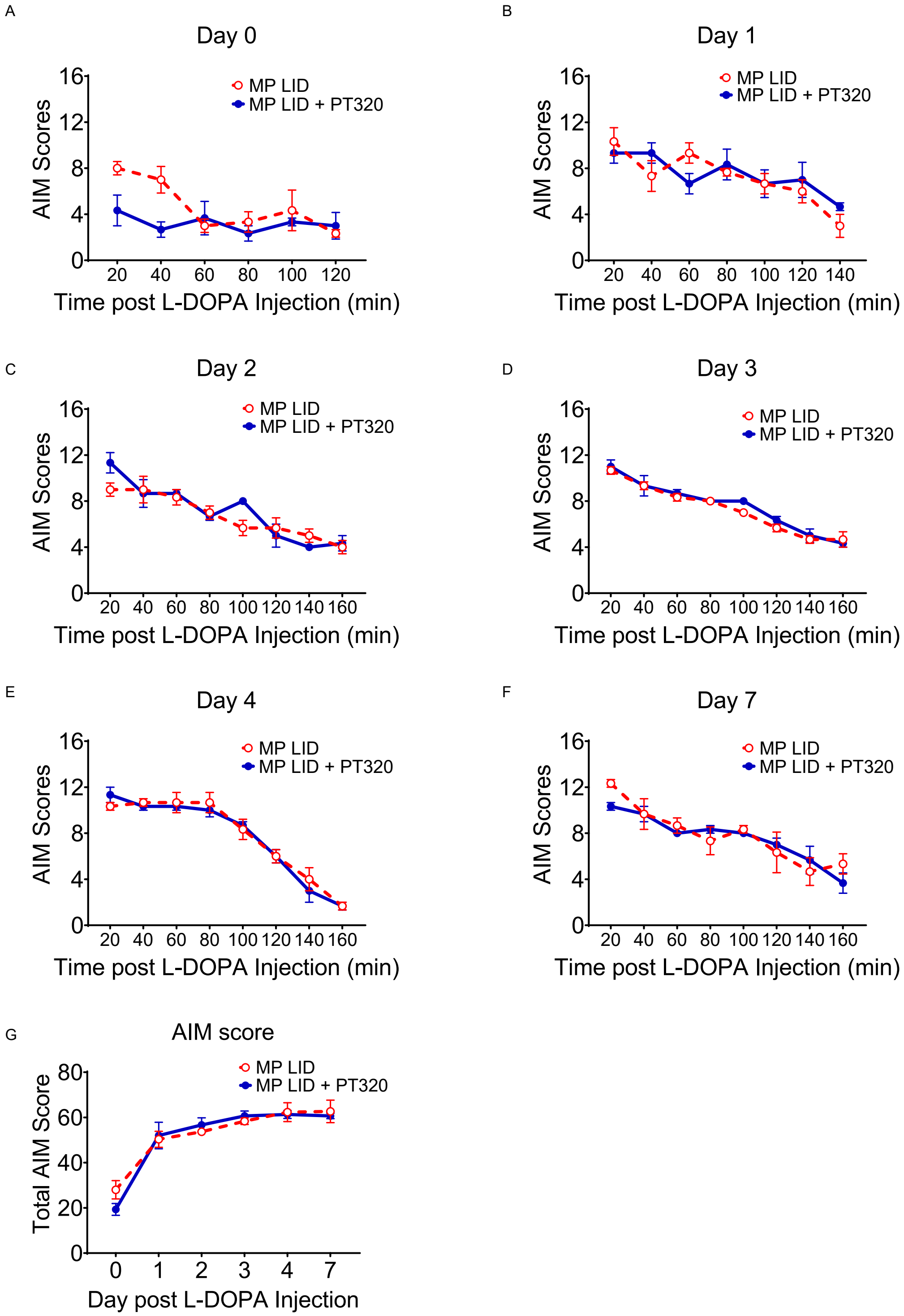

Supplementary Figure S4
